# Studies of the Variability of Biologically Active Compounds and Antioxidant Activity in Organically, Biodynamically, and Naturally Grown and Fermented Fireweed (*Chamerion angustifolium* (L.) Holub) Leaves

**DOI:** 10.3390/plants12122345

**Published:** 2023-06-16

**Authors:** Marius Lasinskas, Elvyra Jariene, Jurgita Kulaitiene, Nijole Vaitkeviciene, Erika Jakiene, Dominika Skiba, Ewelina Hallmann

**Affiliations:** 1 Department of Plant Biology and Food Sciences, Agriculture Academy, Vytautas Magnus University, Donelaicio St. 58, 44248 Kaunas, Lithuania; elvyra.jariene@vdu.lt (E.J.); jurgita.kulaitiene@vdu.lt (J.K.); nijole.vaitkeviciene@vdu.lt (N.V.); navickaiterika08@gmail.com (E.J.); 2 Department of Plant Production Technology and Commodities Science, University of Life Sciences in Lublin, 20-950 Lublin, Poland; dominika.skiba@up.lublin.pl; 3 Department of Functional and Organic Food, Institute of Human Nutrition Sciences, Warsaw University of Life Sciences, Nowoursynowska 15c, 02-776 Warsaw, Poland; ewelina_hallmann@sggw.edu.pl; 4 Bioeconomy Research Institute, Agriculture Academy, Vytautas Magnus University, Donelaicio St. 58, 44248 Kaunas, Lithuania

**Keywords:** antioxidant activity, biodynamic, fermentation, fireweeds, phenolic acids

## Abstract

With the growing global demand for organically and biodynamically grown fireweeds, little research is being conducted on them, and little is known about how the different growing systems and the process of solid-phase fermentation changes biologically active substances and antioxidant activity. Our experiment was conducted in 2022 in Jonava district, Safarkos village, Giedres Nacevicienes organic farm (No. SER-T-19-00910, Lithuania, 55°00′22″ N 24°12′22″ E). This study aimed to investigate the influence of various growing systems (natural, organic, and biodynamic) and technological parameters (different duration: 24, 48 and 72 h) of aerobic solid-phase fermentation on the change of flavonoids, phenolic acids, tannins, carotenoids, chlorophylls, and antioxidant activity. High-performance liquid chromatography (HPLC) for polyphenols, carotenoids, and chlorophylls as well as the spectrophotometric method for antioxidant activity determinations were used. The results of the study showed that different growing systems (natural, organic, and biodynamic) and solid-phase fermentation had a significant effect on the quantitative composition of biologically active substances in the leaves of the fireweeds. According to these data, it would be possible to recommend fermented fireweed leaves grown organically as a source of polyphenols (especially: phenolic acids and flavonoids), leaves grown biodynamically as a source of carotenoids (exceptionally: lutein and beta-carotene) and chlorophyll, and leaves grown naturally for better antioxidant activity.

## 1. Introduction

The growing concern over the spread of chemical inputs in the agri-environment, and their economic and social impacts have prompted many farmers and consumers to look for alternative methods and systems in order to make agriculture more sustainable. Alternative farming systems encompass biological, biodynamic, organic, and low-cost farms. Such farms produce adequate food of high quality, are environmentally safe, protect the soil resource base, and are both profitable and socially correct [1]. Many studies have shown that organic farming, which avoids the use of synthetic fertilizers and pesticides, may lead to increased soil biodiversity and biological activity in soils when compared to conventional farming [2]. Biodynamic farming, as a specific form of organic farming, has also been reported to sustain better soil quality than conventional farming practices. In addition to different farming systems, factors such as plant species [3], soil type [4], and tillage may also influence biological soil characteristics.

According to some authors, soil that has been managed organically or biodynamically has more microorganisms when managed than are found in conventional farming. These soil microorganisms produce many compounds that help plants, including substances that combine with soil minerals and make them more available to plant roots. The presence of soil microorganisms at least partially explains the trend showing a higher mineral content of organic food crops, which is associated with a higher quality of the plant material [5,6].

Recently, researchers have been particularly interested in biologically active compounds characterized by antioxidant activity. Fireweed (*Chamerion angustifolium* (L.) Holub) is one of the best-known medicinal plants and is used in traditional medicine worldwide. Flavonoids and ellagitaninns, such as oenothein B, are one of the most important biologically active compounds present in fireweed extracts [7]. Therefore, it is very important to know both the composition and the pharmacological properties of fireweed leaves and extracts. Nevertheless, the availability of comprehensive research targeting this subject is very limited. It is necessary to compare different farming systems (organic and biodynamic) to evaluate the potential benefits of biodynamic farming for the quality of fireweed leaves.

Fireweed leaves are usually collected at the beginning of July in the stage of the full flowering of plants, when the plant synthesizes the most biologically active substances [8]. The strong antioxidant effect has been attributed to the high content of ellagitannins and especially oenothein B [9]. Evaluation of the radical-scavenging activity of fireweeds collected during the growing season concerning their flavonoid content has been studied by Maruška et al. [10].

Many people around the world consume green or black tea, but other fermented teas made from fireweed leaves are gaining interest. One of the methods of making functional fireweed tea and also improving the quantitative and qualitative composition of fireweed leaves and other products is the use of solid-phase fermentation technology. During this process, biochemical reactions take place inside the cells and there is also strong activity of microorganisms and enzymes [11].

It seems that not only the high content of bioactive compounds in plants but also the level of their availability in infusions is essential. Indeed, it is very important to find a way to increase the bioavailability of biologically active compounds in plants. One of the ways to modulate biologically active compounds and their bioavailability in fireweed leaves today is to use solid-phase fermentation.

The application of the biodynamic system as well as a comparison of its efficiency with organic systems has not yet been investigated. In addition, no attempts have focused on the effects of different farming systems on the accumulation of bioactive compounds in fireweed leaves. It is crucial to match the best agronomic methods in order to maximize the contents of bioactive compounds with health-promoting properties.

The results of our research could help farmers to choose a more valuable way of growing fireweeds using the principles of biodynamic farming, and producers of health products and healthy foods could produce high-quality products and dietary supplements with fireweed leaf extracts.

## 2. Results and Discussion

The obtained results showed that organic fireweeds are characterized by the highest concentration of total polyphenols (4189.80 mg 100 g^−1^ D.M.) (Table 1). The used levels of fermentation of the fireweed leaves affect the diminishing levels of total polyphenols in the fireweed leaves. A significant concentration of total polyphenols was observed in control plants. After 24 h of fermentation, the concentration of the total polyphenols decreased by about 16.3%. The longer fermentation process still diminishes the concentration of total polyphenols up to the next 35.3% compared to 24 h. A significant concentration of total phenolic acids and total flavonoids was observed in organic samples (3064.99 mg 100 g^−1^ D.M. and 1124.81 mg 100 g^−1^ D.M., ly). The biodynamic and natural experimental combination were not statistically significant. Similar to the previous study, we observed that the fermentation process significantly diminishes the total phenolic acids and total flavonoids in the fireweed leaves. The lowest concentration that was observed in both bioactive compounds groups was after 48 h of the fermentation process. Total carotenoid concentration was the highest in the natural and biodynamic combination (60.42 mg 100 g^−1^ D.M. and 66.25 mg 100 g^−1^ D.M., respectively), and the lowest content of the total carotenoids we observed was in the organic fireweed leaves. The first step of fermentation decreased the content of total carotenoids in leaves, but we did not observe differences after 24 h and 48 h in fireweed leaves. The significant lowest concentration of the total chlorophylls was measured in organic fireweed samples. Biodynamic samples are characterized by the significantly higher concentration of total chlorophyll content (274.84 mg 100 g^−1^ D.M.). The fermentation process reduces the total chlorophyll content in fireweed leaves, though: after 24 h, the concentration was 36.9% lower compared to the control samples, and after 48 h, we still observed progress diminishing by 6.3% (Table 1).

In the case of interaction, we observed that the fermentation process negatively affects the concentration of the total polyphenols in fireweed leaves (Table 2). In all experimental combinations, the decrease in the total polyphenols was observed. The highest diminishing level was observed in the organic samples. After 24 h, it was 17.9%, and after 48 h, it was 56.1% compared to the control plants. The lowest decrease was observed in the combination of biodynamic samples. After 24 h, it was 16.1%, and after 48 h, it was 12.5% compared to the control plants. A similar relationship was observed in the case of changes in the content of phenolic acids. The greatest decrease was observed in the organic samples. The smallest amount of phenolic acids was lost in biodynamic samples after 48 h, and it was only 15.5% compared to the control samples. It is worth pointing out that in the case of total flavonoids, much higher decreasing levels were observed after 24 h compared to 48 h, but this was only in the organic and biodynamic samples. In the natural combination, much more diminishing was observed after 48 h (15.4%) than after 24 h (8.4%). In the case of carotenoids, only in the first 24 h was there a decrease in bioactive compounds: we observed a 4.5%, 8.6%, and 6.6% decrease, respectively, for biodynamic, organic, and natural samples. A longer fermentation period was favorable for both the organic and the natural samples. In these cases, an increase in the concentration of total carotenoids was observed in the fermented fireweed samples. In the case of total chlorophylls, much more decreasing was observed in the first 24 h of fermentation. The next 24 h was favorable only for natural samples. In that case, the concentration of the total chlorophylls increased by about 9.8% compared to the samples after 24 h of fermentation (Table 2).

To our best knowledge, no previous research has been published on the influence of production systems on the content of secondary metabolites, such as the phenolic compounds and the carotenoids of fireweed leaves. According to some researchers, any significant stressor in the agricultural environment affects plant metabolism as well as the accumulation of phenolic compounds and carotenoids. The accumulation of secondary metabolites in plant tissues may also increase if there is a limited supply of plant nutrients (especially nitrogen) available during cultivation. Therefore, by using various cultivation and fertilization techniques, stress factors can be avoided or generated [12,13].

According to the study conducted by Vaitkeviciene et al. [14], biodynamically grown potato tubers contained higher levels of total phenolics and total phenolic acids than organic and conventionally grown tubers. These researchers also established that the production system influenced the content of total carotenoids in the potato tubers. The higher levels of this compound are identified in biodynamic potatoes compared to organic potatoes. The content of organic matter in the soil could also influence the epoxidation and de-epoxidation processes of carotenoids in the xanthophyll cycle [15].

The significantly highest concentration of gallic acid was observed in organic and natural leaves of fireweeds (31.22 mg 100 g^−1^ D.M. and 32.09 mg 100 g^−1^ D.M., respectively) (Table 3). The fermentation process after 48 h finally decreased the level of gallic acid, but it was worth observing that in the first 24 h, the concentration of gallic acid significance increased. In the case of chlorogenic acid, the highest and most significant concentration of that compound was observed in the natural samples (47.37 mg 100 g^−1^ D.M.). As previous fermentation decreased the level of chlorogenic and *p*-coumaric acids, we did not observe differences between 24 h and 48 h of fermentation. The highest concentration of *p*-coumaric and ellagic acids (208.41 mg 100 g^−1^ D.M. and 2790.44 mg 100 g^−1^ D.M., respectively) was observed in organic fireweed leaves. Fermentation for 24 h did not affect the concentration of ellagic acid, but it decreased the concentration significantly over the next 24 h. Benzoic acid was identified in the highest concentration (6.57 mg 100 g^−1^ D.M.) in the natural samples. The fermentation process significantly decreased the level of benzoic acid (Table 3).

In the case of interaction, we observed that the fermentation process positively affects only gallic acid concentration (Table 4). After 48 h, the concentration of gallic acid was significantly higher. This situation was observed only in biodynamic samples. In the organic sample, 24 h decreased the level of gallic acid, and, finally, the next 24 h increased it up to the starting level. In the case of chlorogenic and p-coumaric acids, both biodynamic and organic samples reacted similarly. After 24 h, we observed a decrease in chlorogenic acid, but longer fermentation yielded more positive results. Only natural samples showed both 24 h and 48 h negative reactions for the fermentation process. In the case of ellagic acid, we observed a decrease in its content after 24 h and even further after 48 h. The greatest decrease in the content was observed in the organic samples. The reaction of benzoic acid was also extremely interesting. The greatest decrease was observed in biodynamic and ecological samples after 24 h of fermentation (Table 4).

In all samples, seven flavonoids were identified and quantified. Among the tested samples, significantly more oenothein B (937.49 mg 100 g^−1^ D.M.) was found in the samples from organic production (Table 5). There were no significant differences between the biodynamic and natural samples. The fermentation process had a negative effect on the content of oenothein B in fireweed leaves. After 48 h of fermentation, samples with the lowest content of this compound were obtained compared to the control samples.

In the case of quercetin-3-O-rutinoside, it was found that the organic fireweed leaves contained the highest concentration of this compound (79.19 mg 100 g^−1^ D.M.). As with the previously discussed oenothein B, there were no differences in quercetin-3-O-rutinoside content between the biodynamic and the natural samples. It seems that to obtain a product with a high concentration of quercetin-3-O-rutinoside, fireweed leaves should be fermented for a short time. After 24 h, a significant increase in rutin concentration was observed, and after another 24 h, its content decreased significantly. Organic samples were characterized by a higher concentration of myricetin (14.81 mg 100 g^−1^ D.M.) compared to natural and biodynamic ones. As we pointed out previously, only a short fermentation time should be used to obtain the product with a high myricetin content. In the case of luteolin, organic samples contained significantly more of this compound. The best plant material is not fermented fireweed leaves. After the fermentation, we observed a decrease in luteolin in all samples. Biodynamic samples were characterized by a higher concentration of quercetin (1.68 mg 100 g^−1^ D.M.) compared to the rest of the experimental combinations. As was the case previously, the best method was not fermented samples. In organic samples, concentrations of quercetin-3-O-glucioside and kaempferol were the highest (88.38 mg 100 g^−1^ D.M. and 1.76 mg 100 g^−1^ D.M., respectively) and statistically significant. After short-term fermentation, we observed a significant increase in quercetin-3-O-glucoside and kaempferol. After the next 24 h, the concentration of these flavonoids returned to the initial value (Table 5).

The 24 h fermentation process contributed to a decrease in oenothein B content in all of the tested samples, but the greatest decrease was observed in the organic samples (Table 6). The next 24 h of fermentation also reduced the content of this compound, but the greatest decrease was observed in the natural samples (15.2%) and the smallest in the organic samples (3.6%). In the case of the organic and biodynamic samples as well as the content of quercetin-3-O-rutoside, the first 24 h of fermentation contributed to an increase in the content of this compound in the tested samples. This phenomenon was not observed in natural samples. After 48 h, significantly more rutin was found in the biodynamic samples (next increasing by about +1.9%) compared to the short fermented samples. It is worth noting that in the organic samples after 48 h, the highest decrease in rutin content was observed in comparison with all of the tested samples. Similarly, as reported previously, the same situation was observed with myricetin. The fermentation process was not dedicated to luteolin concentration. Both short (24 h) and long (48 h) fermentation decreased the concentration of these compounds in all experimental samples. In the case of quercetin, we observed that only in the natural samples after short fermentation was the concentration of quercetin increased. In the case of quercetin-3-O-glucoside and kaempferol, only short-time fermentation (24 h) seems preferable for the highest concentration in all experimental samples of fireweed leaves (Table 6).

The highest and statistically significant concentration of lutein and beta-carotene (35.59 mg 100 g^−1^ D.M. and 15.90 mg 100 g^−1^ D.M., respectively) was found in biodynamic samples (Table 7). In the case of zeaxanthin, the best experimental combination (16.19 mg 100 g^−1^ D.M.) was natural. The fermentation process decreased the level of lutein and beta-carotene in fireweed samples. It seems that long-term fermentation positively affects the concentration of zeaxanthin. After 48 h, we observed the highest concentration in fireweed leaves compared to the rest of the experimental combinations. The content of carotenoids was highly variable under the influence of the fermentation process carried out. Only in the biodynamic samples and after using a short fermentation time did we observe an increase in lutein concentration in the tested samples. In the organic and natural combination, the lutein content decreased after 24 h of fermentation.

However, the further process of biodynamic sampling ended with a decrease in the concentration of lutein, and in the case of organic and natural samples, the opposite phenomenon was observed. In the case of beta-carotene, both short and long fermentation were affected by beta-carotene decreasing, excluding the natural samples. In these samples, a small increase in beta-carotene concentration (+2.7%) was observed (Table 7 and Table 8).

Organic samples were characterized by the highest and most statistically significant concentration of chlorophyll *a*, while biodynamics contained the highest level of chlorophyll *b*. The fermentation process negatively affected all samples in the case of both chlorophylls (*a* and *b*). In the biodynamic and organic samples, we observed the decrease in chlorophylls *a* and *b* after 24 h and 48 h of fermentation. Only in the natural samples did long-term fermentation show a slight increase in both forms of chlorophyll (Table 8).

The process of solid-phase fermentation and enzymes produced during the metabolism of microorganisms (lactic acid bacteria and yeast), such as polyphenol oxidase, etc., breaks down the macromolecular compounds contained in the leaves of the fireweeds into lower molecular weight substances and secondary metabolite products [16], and it can thus decrease the quantities of some compounds.

On the other hand, the crushing and pressing of the leaves during solid-phase fermentation can enhance the degradation processes of the cell walls and thus improve the diffusion of biologically active substances from the inner parts of the cells, which leads to a more efficient extraction of compounds. There is some data that some solid-phase fermentation parameters could activate the process of the accumulation of some bioactive compounds in the fireweed leaves [17,18].

**Figure 1:**
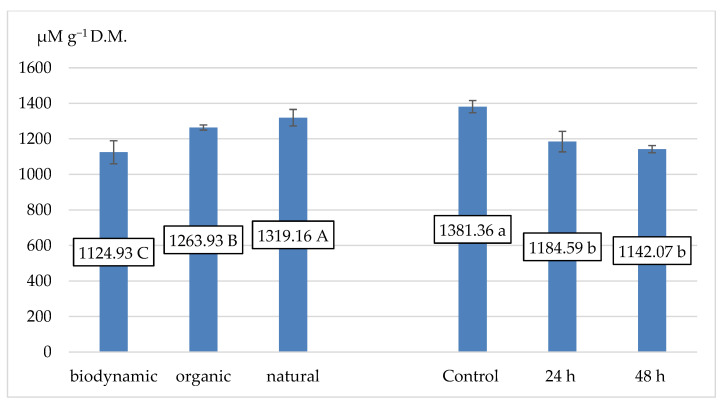
The mean value for antioxidant activity (in µM Trolox eq. g^−1^ D.M.) for different fireweed leaves. Means on bars followed by the same letter are not significantly different at the 5% level of probability (*p* < 0.05).

The obtained results showed that natural samples were characterized by the significant and highest antioxidant activity (1319.16 µM Trolox eq. g^−1^ D.M.). Organic samples were classified before biodynamics. The fermentation process negatively affected the parameters of the antioxidant activity of fireweed leaves. The highest activity was shown by unfermented samples, and the lowest was by samples after 48 h of fermentation (Figure 1).

**Figure 2:**
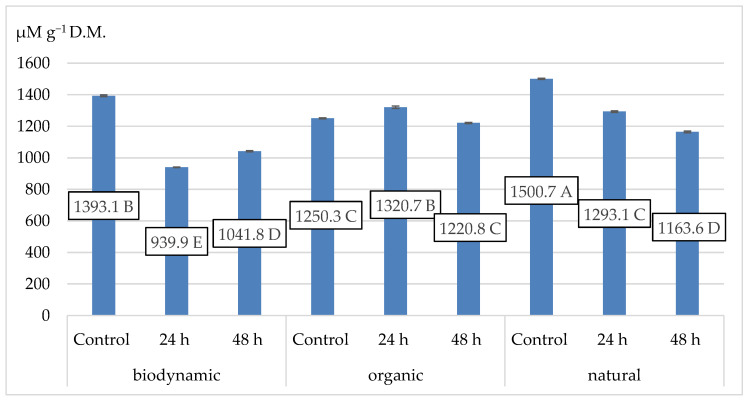
The antioxidant activity (in µM Trolox eq. g^−1^ D.M.) for different fireweed leaves according to the fermentation process. Means on bars followed by the same letter are not significantly different at the 5% level of probability (*p* < 0.05).

Each of the tested organic, biodynamic, and natural samples reacted differently to the fermentation time. The 24-h short fermentation process reduced the activity of fireweed leaves in biodynamic and natural samples, but not in the organic ones. After further hours of fermentation, an increase in antioxidant activity was observed in the organic and the natural samples but not in the biodynamic ones (Figure 2).

Data from the scientific literature on how different production systems affect the antioxidant activity in plants’ raw materials differs. In the study conducted by Heimler et al. [19], higher antioxidant activity was identified in Batavia lettuce grown under biodynamic production systems. On the other hand, Italian scientists evaluated the antioxidant activity of Albana and Lambrusco grape berries cultivated in conventional, biodynamic, and organic systems, and they found no significant differences [20].

## 3. Materials and Methods

### 3.1. Field Experiment

**Figure 3:**
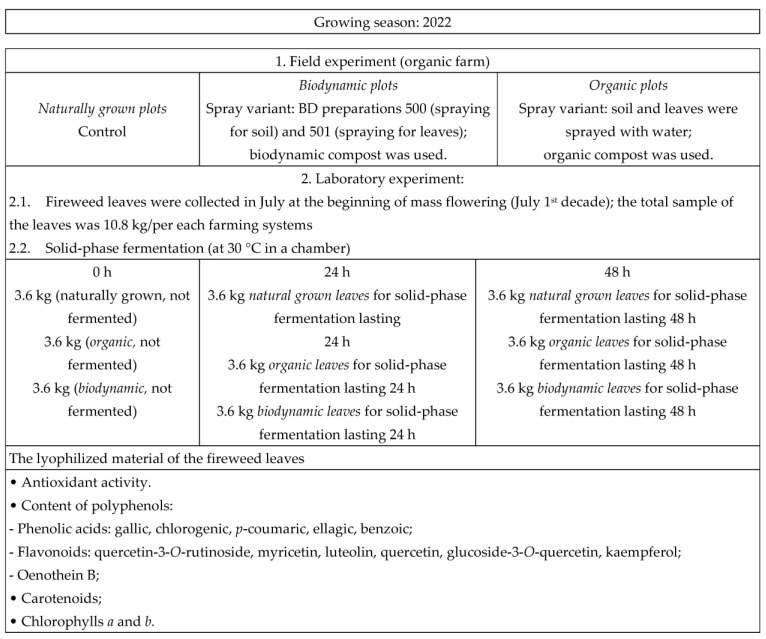
Effect of farming systems on the accumulation of biologically active compounds of fermented fireweed leaves: experimental design.

The field experiment was conducted in 2022 in Jonava district, Safarkos village, Giedres Nacevicienes organic farm (No. SER-T-19-00910, Lithuania, 55°00′22″ N 24°12′22″ E). Organic fields were managed according to this cultivation system for the last 10 years, with a transition to biodynamic farming in the past year. The perennial fireweeds in the organic farm are grown for the fourth year. Part of the field was left for fireweeds to grow naturally. The total area of the experimental plot was 20 acres (2000 m^2^). The experimental design is presented in Figure 3.

In the field experiment, fireweed plants were grown according to two different farming systems: organic and biodynamic, and they were compared with natural growing (control). The distance between rows was 70 cm, and the distance between plants in a row was 30 cm. The soil in the spaces between the rows was fertilized with organic compost (25 t ha^−1^) or with biodynamic compost (25 t ha^−1^) approximately two weeks before the beginning of the vegetation of the plants (in the middle of June).

Organic experimental fields were sprayed with water in the middle of June, and leaves of the fireweed were sprayed with water two times during the vegetation period—in the morning at the stage of leaf formation at the end of June and at the beginning of plants mass flowering (1st July decade).

Biodynamic experimental fields were sprayed with biodynamic (BD) preparation 500 in May (2nd decade) at a concentration of 1% solution (200 L ha^−1^), and leaves of the fireweed were sprayed with BD preparation 501 0.5% solution twice during the vegetation period—in the morning at the stage of leaf formation middle of June and at the beginning of mass flowering of the plants (1st July decade) (200 L ha^−1^).

Plant protection products against diseases and vermin were not used. The soil and plants of the fireweed in the biodynamic system were sprayed with BD preparations according to the methodologies used in European biodynamic farms [21]. Biodynamic compost and BD preparations 500 and 501 used in the experiment were purchased from a Demeter-certified farm (CvW KG, Internationale Biodynamische Praparatezentrale, Germany).

The variants were rendered in three replications. From each replication, 10.8 kg of herbal raw material as collected for testing. Test boxes with and without biodynamic additives were established at the same sampling site (farm).

Laboratory experiment—solid-phase aerobic fermentation:

1. Control (not fermented).

2. Fermented (24 h).

3. Fermented (48 h).

### 3.2. Plant Material Preparation and Solid-Phase Aerobic Fermentation Process

The raw material was randomly collected from three replications (field plots) in each agronomic system in July at the beginning of mass flowering (1st July decade). The total sample of the leaves was 10.8 kg/per each farming system:

✓ Naturally grown (control): 3.6 kg (not fermented) for control, 3.6 kg for solid-phase fermentation lasting 24 h, and 1.2 kg for 48 h fermentation.
✓ Organically grown fireweed: 3.6 kg (not fermented) for control, 3.6 kg for solid-phase fermentation lasting 24 h, and 3.6 kg for 48 h fermentation.
✓ Biodynamically grown fireweed: 3.6 kg (not fermented) for control, 3.6 kg for solid-phase fermentation lasting 24 h, and 3.6 kg for 48 h fermentation.

Control (0 h)—unfermented leaves but stored for the intended time.

In the solid-phase fermentation, fresh fireweed leaves were cut with special plastic knives, and the resulting raw material was divided into three subsamples of 1.2 kg. The mass ready for fermentation was rigidly pressed into glass containers and covered with an air-passing lid. The fermentation process took place at 30 °C in the chamber for 24 h and 48 h.

### 3.3. Laboratory Analyses

#### 3.3.1. Preparation of Fireweed Leaves for Fermentation

After fermentation, the raw materials were frozen at −35 °C, then lyophilized in a ZIRBUS sublimation dryer 3 × 4 × 5 (ZIRBUS Technology, Bad Grund, Germany). The lyophilized leaves were milled to powder using a Grindomix GM 200 laboratory mill (Retsch GmbH, Haan, Germany) for further analyses.

#### 3.3.2. Polyphenols Identification and Quantification

100 mg of freeze-dried plant material were weighed, 5 mL of 80% methanol was added, vortexed (Micro-Shaker 326 M, Premeo, Poland), incubated in an ultrasonic bath (10 min, 30 °C, 5500 Hz), and then centrifuged (10 min, 3780× *g*, 5 °C). The obtained supernatants were centrifuged again (5 min, 31.180× *g*, 0 °C), and then 900 µL of the clear supernatants were transferred to HPLC vials and analyzed. Identification and separation of phenolic compounds (such as phenolic acids, flavonoids, and flavonols) were carried out by the HPLC method using Shimadzu kit (USA Manufacturing Inc., Raleigh, NC, USA, including two LC-20AD pumps, CBM-20A controller, SIL-20AC column oven, UV/Vis SPD-20 AV spectrometer). The phenolic compounds were separated on a Synergi Fusion-RP 80i Phenomenex column (250 × 4.60 mm), with a flow rate of 1 mL min^−1^. Two gradient phases (acidified with ortho-phosphoric acid, pH 3.0) were used: 10% (*v*:*v*) acetonitrile and ultrapure water (phase A) and 55% (*v*:*v*) acetonitrile, and ultrapure water (phase B). The total analysis time was 38 min, with the following phase-time program: 1.00–22.99 min, 95% phase A and 5% phase B; 23.00–27.99 min, 50% phase A and 50% phase B; 28.00–28.99 min, 80% phase A and 20% phase B; and 29.00–38.00 min, 95% phase A and 5% phase B. The wavelengths were λ = 250 nm for flavonols and λ = 370 nm for phenolic acids. The phenolic compounds were identified using 99.9% pure standards (Sigma-Aldrich, Poland) and the specified analysis times for the standards.

#### 3.3.3. Carotenoids Identification and Quantification

The freeze-dried fireweed tissue (100 mg) were mixed with acetone (5 mL). Samples were then ultrasonicated (15 min at the temperature of 0 °C) and centrifuged (6000 rpm, 10 min, 0 °C). Following this step, the supernatant was collected into HPLC- vials. The Max-RP 80A column (250 × 4.6 mm) was used for the compounds separation. The injection volume was 100 μL and detection was performed under the wavelength of 445 and 450 nm for 18 min. The quantification of the compounds was based on external standards.

#### 3.3.4. Chlorophylls Identification and Quantification

Chlorophylls (*a* and *b*) were measured by the HPLC (Polish agent Shimpol, Warsaw, Poland) method described by Ponder and Hallmann (2020) [22]. Sample preparation included extraction of 100 mg of a freeze-dried sample with 100% acetone using an ultrasonic cold bath (10 min, 0 °C, 5.5 kHz). Samples were then centrifuged (10 min, 6000 rpm, 0 °C). One milliliter of supernatant was transferred into an HPLC vial. The wavelength used for detection was 445–450 nm. The concentrations of the chlorophylls were calculated using standard curves and the sample dilution coefficient, and they were presented in mg per 100 g of dry matter.

#### 3.3.5. Antioxidant Activity

To measure the antioxidant activity, the colorimetric spectrophotometric method with ABTS^+•^ (2,2′-azino-bis (3-ethylbenzothiazoline-6-sulfonic acid) cation radicals was used. According to the modified procedure by Re et al. [23], 0.0384 g of ABTS radical reagent was dissolved in 5.0 mL of deionized water, then 5.0 mL of aqueous potassium persulfate (K2S2O8) (prepared by dissolving 0.026 g in 20 mL of deionized water) was added and the ABTS^+•^ radical solution (10 mL) was incubated at 21 °C in the dark for 12 h and finally diluted with a PBS solution (phosphate-buffered saline) in order to obtain a blank sample absorbance of 0.700 ± 0.02 at λ = 734 nm. Measurements were made in water extracts of tested samples, and were prepared as described above. To determine the antioxidant activity, 1.5 mL (diluted with PBS solution) was dispensed into 10 mL glass test tubes, 3.0 mL of ABTS^+•^ cation radical solution (with a predetermined absorbance of 0.700 ± 0.02) was added, incubated at 21 °C for 6 min, and then the absorbance was measured in a spectrophotometer (UV–VIS UV-6100A, Metash Instruments Co., Ltd., Shanghai, China) at a wavelength λ = 734 nm. The determination was performed in nine independent repetitions, and after accounting for the applied dilutions, the results were calculated on the basis of the calibration curve (y = −5.6017 × 0.7134, R^2^ = 0.9998) for Trolox as a reference substance and expressed as µM TEAC 100 g^−1^ D.M.

### 3.4. Statistical Analysis

The obtained results were analyzed using the statistical program Statgraphics Centurion 15.2.11.0 (StatPoint Technologies, Inc., Warranton, VA, USA). The tables show the average values of *9* individual measurements for the production system (biodynamic, organic, and natural) and, for the two fermentation times, 24 h and 48 h compared to the unfermented control. A two-way analysis of variance with Tukey’s test was performed, and differences between the groups at the level of *p* < 0.05 were considered statistically significant. The standard error (SE) was also given for each mean value presented in the tables.

## 4. Conclusions

The discussed results showed that different growing systems and solid-phase fermentation affected the amounts of biologically active and antioxidant activity in the leaves of fireweeds. The used levels of fermentation of the fireweed leaves affected the diminishing of total polyphenols, total carotenoids, total chlorophylls, and antioxidant activity. According to these data, it would be possible to recommend unfermented fireweed leaves grown organically as a source of polyphenols, leaves grown biodynamically as a source of carotenoids and chlorophylls, and naturally grown leaves for better antioxidant activity.

In summary, solid-phase fermentation reduced the amounts of biologically active and antioxidant activity in fireweed leaves. Based on the data of this study, in an organic growing system, the food and pharmacy industry could produce fireweed leaves as functional food products with higher polyphenols; the biodynamically grown leaves of fireweeds could be very useful for people due to high carotenoids and chlorophyll content; and naturally grown fireweeds could be useful due to their superior antioxidant properties.

It must be considered that the proposed method will give the assumed results with fireweed (*Chamerion angustifolium* (L.) Holub) leaves or plants with such a composition. This experiment is a field laboratory one, and we will continue this experiment for the next year in order to confirm our findings.

## Figures and Tables

**Table 1:** The mean value for the content of total polyphenols, carotenoids, and chlorophylls (in mg 100 g^−1^ D.M.) in fireweed according to different production systems and duration times.

Mean Values	Total Polyphenols	Total Phenolic Acids	Total Flavonoids	Total Carotenoids	Total Chlorophylls
Biodynamic	2737.52 ± 117.0 ^1^ B ^2^	1892.28 ± 76.7 B	845.24 ± 43.0 B	66.25 ± 2.2 A	274.84 ± 21.3 A
Organic	4189.80 ± 519.0 A	3064.99 ± 447.5 A	1124.81 ± 85.0 A	55.52 ± 0.8 B	200.72 ± 16.1 C
Natural	2351.30 ± 114.3 B	1498.79 ± 85.2 B	852.51 ± 29.7 B	60.42 ± 1.1 A	248.72 ± 24.4 B
Control	3901.70 ± 444.7 a	2752.05 ± 368.1 a	1149.65 ± 77.0 a	63.33 ± 2.4 a	326.41 ± 15.0 a
24 h	3264.02 ± 347.0 a	2372.39 ± 317.7 a	891.62 ± 35.0 b	59.25 ± 2.0 b	205.67 ± 8.5 b
48 h	2112.89 ± 70.4 b	1331.61 ± 70.8 b	781.28 ± 4.0 c	59.61 ± 0.7 b	192.21 ± 3.8 b
*p*-Value					
Production system	<0.0001	<0.0001	<0.0001	<0.0001	<0.0001
Duration	<0.0001	<0.0001	<0.0001	0.049	<0.0001

^1^ Data are presented as the mean ± SE (standard error) with ANOVA *p*-value. ^2^ Means in a column followed by the same letter are not significantly different at the 5% level of probability (*p* < 0.05).

**Table 2:** The content of total polyphenols, carotenoids, and chlorophylls (in mg 100 g^−1^ D.M.) in fireweed according to different production systems and duration times.

Production System	Duration	Total Polyphenols	Total Phenolic Acids	Total Flavonoids	Total Carotenoids	Total Chlorophylls
Biodynamic	Control	3191.95 ± 18.2 ^1^ b ^2^	2165.90 ± 17.5 b	1026.05 ± 2.1 b	70.79 ± 4.9 a	363.77 ± 3.01 a
	24 h	2678.13 ± 28.2 c	1902.70 ± 25.1 b	775.43 ± 4.8 d	67.58 ± 0.6 a	240.18 ± 4.8 b
	48 h	2342.48 ± 7.7 d	1608.24 ± 9.3 d	734.23 ± 1.7 d	60.38 ± 0.4 b	220.56 ± 6.7 c
Organic	Control	5765.08 ± 86.4 a	4292.75 ± 85.4 a	1472.33 ± 17.9 a	58.79 ± 0.5 d	264.14 ± 6.4 b
	24 h	4729.96 ± 7.8 a	3700.99 ± 10.6 a	1028.97 ± 18.2 b	53.76 ± 0.6 d	188.63 ± 6.5 e
	48 h	2074.34 ± 20.6	1201.22 ± 6.7 e	873.12 ± 16.6 c	54.01 ± 0.4 d	149.40 ± 1.1 f
Natural	Control	2748.09 ± 54.4 c	1797.51 ± 51.2 c	950.58 ± 5.5 bc	60.42 ± 0.7 b	351.31 ± 1.5 a
	24 h	2383.95 ± 5.1 d	1513.49 ± 7.1 de	870.46 ± 2.1 c	56.42 ± 0.2 c	188.18 ± 2.9 e
	48 h	1921.86 ± 15.0 e	1185.36 ± 12.3 f	736.50 ± 9.6 d	64.43 ± 0.3 a	206.67 ± 3.2 d
*p*-Value						
Production × duration		<0.0001	<0.0001	<0.0001	0.0125	<0.0001

^1^ Data are presented as the mean ± SE (standard error) with ANOVA *p*-value. ^2^ Means in a column followed by the same letter are not significantly different at the 5% level of probability (*p* < 0.05).

**Table 3:** The mean value for the individual identified phenolic acids (in mg 100 g^−1^ D.M.) in fireweed according to different production systems and duration times.

Mean Value	Gallic	Chlorogenic	*P*-Coumaric	Ellagic	Benzoic
Biodynamic	26.34 ± 1.4 ^1^ B ^2^	34.29 ± 1.8 B	80.33 ± 2.3 B	1746.66 ± 73.6 AB	4.66 ± 2.0 B
Organic	31.22 ± 0.7 A	32.83 ± 1.0 B	208.41 ± 13.3 A	2790.44 ± 440.5 A	2.09 ± 0.4 C
Natural	32.09 ± 3.2 A	47.37 ± 2.8 A	39.66 ± 3.2 C	1373.09 ± 76.3 B	6.57 ± 0.7 A
Control	30.77 ± 2.6 a	45.14 ± 2.8 a	135.29 ± 30.7 a	2532.13 ± 341.0 a	8.71 ± 1.4 a
24 h	31.74 ± 1.6 a	36.30 ± 3.0 b	93.31 ± 19.4 b	2208.17 ± 301.8 a	2.88 ± 0.9 b
48 h	27.14 ± 2.0 b	33.06 ± 0.9 b	99.80 ± 8.1 b	1169.88 ± 62.2 b	1.73 ± 0.6 c
*p*-Value					
Production system	<0.0001	<0.0001	<0.0001	<0.0001	<0.0001
Duration	<0.0001	<0.0001	<0.0001	<0.0001	<0.0001

^1^ Data are presented as the mean ± SE (standard error) with ANOVA *p*-value. ^2^ Means in a column followed by the same letter are not significantly different at the 5% level of probability (*p* < 0.05).

**Table 4:** The content of individual identified phenolic acids (in mg 100 g^−1^ D.M.) in fireweed according to different production systems and duration times.

Production System	Duration	Gallic	Chlorogenic	*P*-Coumaric	Ellagic	Benzoic
Biodynamic	Control	20.66 ± 0.1 ^1^ d ^2^	41.91 ± 0.3 b	89.13 ± 0.9 c	2000.82 ± 18.2 b	13.37 ± 0.04 a
	24 h	28.01 ± 0.1 c	29.84 ± 0.2 e	72.44 ± 1.0 d	1772.05 ± 24.0 c	0.36 ± 0.01 h
	48 h	30.35 ± 0.7 c	31.13 ± 0.4 d	79.43 ± 0.7 d	1467.10 ± 9.2 d	0.23 ± 0.01 i
Organic	Control	32.58 ± 0.5 b	36.74 ± 0.4 c	263.74 ± 1.3 a	3956.20 ± 86.9 a	3.49 ± 004 e
	24 h	28.56 ± 0.1 c	29.93 ± 0.3 e	172.52 ± 3.7 b	3468.14 ± 13.2 a	1.85 ± 0.04 f
	48 h	32.52 ± 0.5 b	31.83 ± 0.2 d	188.96 ± 2.3 b	946.98 ± 7.8 f	0.93 ± 0.10 g
Natural	Control	39.08 ± 0.4 a	56.79 ± 0.3 a	53.00 ± 1.2 e	1639.38 ± 52.8 c	9.26 ± 0.30 b
	24 h	38.65 ± 0.1 a	49.12 ± 0.4 b	34.97 ± 0.5 f	1384.33 ± 6.4 d	6.42 ± 0.04 c
	48 h	18.55 ± 0.2 d	36.21 ± 0.2 c	31.01 ± 0.6 f	1095.57 ± 12.0 e	4.02 ± 0.02 d
*p*-Value						
Production × duration		<0.0001	<0.0001	<0.0001	<0.0001	<0.0001

^1^ Data are presented as the mean ± SE (standard error) with ANOVA *p*-value. ^2^ Means in a column followed by the same letter are not significantly different at the 5% level of probability (*p* < 0.05).

**Table 5:** The mean value for individual identified flavonoids (in mg 100 g^−1^ D.M.) and tannin oenothein B in fireweeds according to different production systems and duration times.

Mean Values	Oenothein B	Quercetin-3-O-Rutinoside	Myricetin	Luteolin	Quercetin	Quercetin-3-O-Glucoside	Kaempferol
Biodynamic	769.43 ± 46.3 ^1^ B ^2^	20.85 ± 1.1 B	8.99 ± 0.5 C	1.707 ± 0.01 A	1.68 ± 0.02 A	40.88 ± 2.0 B	1.70 ± 0.01 B
Organic	937.49 ± 81.7 A	79.19 ±13.6 A	14.81 ± 1.0 A	1.706 ± 0.01 B	1.48 ± 0.03 B	88.38 ± 4.5 A	1.76 ± 0.03 A
Natural	773.52 ± 28.4 B	16.44 ± 0.7 B	13.18 ± 1.4 B	1.707 ± 0.01 A	1.37 ± 0.01 B	44.61 ± 2.2 B	1.68 ± 0.01 B
Control	1039.45 ± 58.8 a	37.12 ± 10.3 b	12.48 ± 1.6 b	1.73 ± 0.01 a	1.57 ± 0.06 a	55.61 ± 9.8 b	1.70 ± 0.02 b
24 h	751.21 ± 14.6 b	55.41 ± 17.0 a	13.82 ± 1.3 a	1.70 ± 0.01 b	1.52 ± 0.04 b	66.20 ± 7.6 a	1.76 ± 0.02 a
48 h	689.79 ± 4.3 c	23.96 ± 1.5 b	10.68 ± 0.4 b	1.69 ± 0.01 b	1.44 ± 0.04 b	52.05 ± 0.5 b	1.68 ± 0.01 b
*p*-Value							
Production system	<0.0001	<0.0001	<0.0001	0.0101	<0.0001	<0.0001	<0.0001
Duration	<0.0001	<0.0001	<0.0001	<0.0001	<0.0001	<0.0001	<0.0001

^1^ Data are presented as the mean ± SE (standard error) with ANOVA *p*-value. ^2^ Means in a column followed by the same letter are not significantly different at the 5% level of probability (*p* < 0.05).

**Table 6:** The content of individually identified flavonoids (in mg 100 g^−1^ D.M.) and tannin oenothein B in fireweeds according to different production systems and duration times.

Production System	Duration	Oenothein B	Quercetin-3-O-Rutinoside	Myricetin	Luteolin	Quercetin	Quercetin-3-O-glucoside	Kaempferol
Biodynamic	Control	964.60 ± 1.7 ^1^ b ^2^	16.24 ± 0.5 d	7.01 ± 0.07 d	1.73 ± 0.003 a	1.77 ± 0.001 a	33.05 ± 0.5 f	1.66 ± 0.001 c
	24 h	690.17 ± 4.9 e	22.92 ± 0.7 c	9.47 ± 0.03 c	1.71 ± 0.001 a	1.67 ± 0.02 b	47.76 ± 0.6 d	1.74 ± 0.005 b
	48 h	653.53 ± 1.3 e	23.38 ± 0.1 c	10.50 ± 0.02 b	1.69 ± 0.001 b	1.61 ± 0.001 b	41.83 ± 0.4 e	1.71 ± 0.002 b
Organic	Control	1282.64 ± 8.6 a	75.79 ± 14.2 b	11.94 ± 0.05 b	1.72 ± 0.001 a	1.60 ± 0.01 b	96.86 ± 2.8 a	1.78 ± 0.002 ab
	24 h	778.95 ± 5.3 d	127.52 ± 0.2 a	18.99 ± 0.30 a	1.70 ± 0.001 b	1.48 ± 0.003 c	98.49 ± 0.6 a	1.84 ± 0.003 a
	48 h	750.88 ± 17.9 d	34.25 ± 0.9 c	13.49 ± 0.50 b	1.70 ± 0.003 b	1.36 ± 0.002 d	69.78 ± 0.6 b	1.66 ± 0.001 c
Natural	Control	871.10 ± 4.6 c	19.32 ± 0.02 d	18.49 ± 0.40 a	1.73 ± 0.001 a	1.34 ± 0.001 d	36.94 ± 1.9 f	1.66 ± 0.002 c
	24 h	784.52 ± 2.2 d	15.77 ± 0.2 de	13.01 ± 0.05 b	1.70 ± 0.001 b	1.40 ± 0.001 c	52.35 ± 0.2 c	1.70 ± 0.001 b
	48 h	664.96 ± 9.4 e	14.24 ± 0.3 e	8.04 ± 0.05 c	1.69 ± 0.001 b	1.35 ± 0.002 d	44.55 ± 0.3 de	1.67 ± 0.001 c
*p*-Value								
Production × duration		<0.0001	<0.0001	<0.0001	0.0002	<0.0001	<0.0001	<0.0001

^1^ Data are presented as the mean ± SE (standard error) with ANOVA *p*-value. ^2^ Means in a column followed by the same letter are not significantly different at the 5% level of probability (*p* < 0.05).

**Table 7:** The mean value for individual identified carotenoids and chlorophylls (in mg 100 g^−1^ D.M.) in fireweed according to different production systems and duration times.

Mean Values	Lutein	Zeaxanthin	Beta-Carotene	Chlorophyll *b*	Chlorophyll *a*
Biodynamic	35.59 ± 2.3 ^1^ A ^2^	14.76 ± 0.9 B	15.90 ± 1.1 A	147.51 ± 7.0 B	127.33 ± 14.4 A
Organic	27.53 ± 0.4 B	15.88 ± 0.4 AB	12.11 ± 0.2 B	127.23 ± 5.5 A	73.49 ± 10.7 C
Natural	32.16 ± 0.7 A	16.19 ± 0.8 A	12.08 ± 0.1 B	156.98 ± 8.3 B	91.74 ± 16.2 B
Control	33.16 ± 2.0 a	14.89 ± 0,7 b	15.28 ± 1.2 a	172.43 ± 5.8 a	153.97 ± 10.1 a
24 h	32.31 ± 2.0 a	14.29 ± 0,4 a	12.65 ± 0.4 b	129.30 ± 2.2 b	76.37 ± 7.6 b
48 h	29.80 ± 0.8 b	17.66 ± 0,2 a	12.15 ± 0.1 b	129.99 ± 2.8 b	62.22 ± 5.1 b
*p*-Value					
Production system	0.0005	0.0001	<0.0001	<0.0001	<0.0001
Duration	n.s.^3^	<0.0001	<0.0001	<0.0001	<0.0001

^1^ Data are presented as the mean ± SE (standard error) with ANOVA *p*-value. ^2^ Means in a column followed by the same letter are not significantly different at the 5% level of probability (*p* < 0.05). ^3^ Not significant.

**Table 8:** The content of individual identified carotenoids and chlorophylls (in mg 100 g^−1^ D.M.) in fireweed according to different production systems and duration times.

Production System	Duration	Lutein	Zeaxanthin	Beta-Carotene	Chlorophyll *b*	Chlorophyll *a*
Biodynamic	Control	37.45 ± 4.9 ^1^ a ^2^	12.84 ± 0.1 e	20.50 ± 0.05 a	176.67 ± 1.9 b	187.11 ± 2.3 a
	24 h	40.48 ± 0.6 a	12.69 ± 0.1 e	14.40 ± 0.04 b	133.62 ± 2.7 d	106.56 ± 2.9 d
	48 h	28.84 ± 0.2 cd	18.75 ± 0.2 a	12.79 ± 0.09 c	132.25 ± 3.3 d	88.31 ± 3.8 e
Organic	Control	28.44 ± 0.2 cd	17.59 ± 0.3 b	12.76 ± 0.04 c	149.50 ± 1.4 c	114.64 ± 4.9 c
	24 h	26.88 ± 0.7 d	14.99 ± 0.1 d	11.89 ± 0.09 d	121.41 ± 1.7 e	67.22 ± 5.3 f
	48 h	27.27 ± 0.4 d	15.06 ± 0.1 c	11.68 ± 0.01 d	110.77 ± 0.1 f	38.62 ± 1.0 g
Natural	Control	33.60 ± 0.5 b	14.23 ± 0.6 cd	12.58 ± 0.01 c	191.14 ± 0.3 a	160.17 ± 1.3 b
	24 h	29.57 ± 0.1 c	15.18 ± 0.1 c	11.66 ± 0.05 d	132.87 ± 1.5 d	55.32 ± 1.4 fg
	48 h	33.29 ± 0.5 b	19.16 ± 0.3 a	11.98 ± 0.04 d	146.95 ± 1.2 c	59.72 ± 2.0 f
*p*-Value						
Production × duration		0.018	<0.0001	<0.0001	<0.0001	<0.0001

^1^ Data are presented as the mean ± SE (standard error) with ANOVA *p*-value. ^2^ Means in a column followed by the same letter are not significantly different at the 5% level of probability (*p* < 0.05).
